# The role of carbonic anhydrase III and autophagy in type 2 diabetes with cardio-cerebrovascular disease

**DOI:** 10.1007/s11011-021-00839-9

**Published:** 2021-10-19

**Authors:** Xiao-Ming Zhang, Ying-Hong Tao, Xiu-Ling Zhou, Xi-Liang Shang, Xiao-Bo Gong, Ying-Chao Liu, Yan-Yan Huang, Gang Chen, Zhong-Yu Yu, Jian-Tao Wang, Zun-Guo Du, Guo-Feng Wu, Yu Zhang, Jing-Chun Guo, Hou-Guang Zhou

**Affiliations:** 1 Geriatrics Department and National Clinical Research Center for Aging and Medicine, Huashan Hospital, and Institutes of Brain Science, Fudan University, Shanghai, 200040 China; 2 Department of Medical Examination Center, Huashan Hospital, Fudan University, Shanghai, 200040 China; 3 Department of Ultrasonics, Huashan Hospital, Fudan Univesity, Shanghai, 200040 China; 4 Department of Sport Medicine, Huashan Hospital, Fudan University, Shanghai, 200040 China; 5 Department of Engineering Mechanics, School of Naval Architecture, Ocean and Civil Engineering, Shanghai Jiao Tong University, Shanghai, 200240 China; 6 Department of Neurosurgery, Provincial Hospital Affiliated to Shandong University, Jinan, 250021 China; 7 Department of Pathology, Huashan Hospital, Fudan University, Shanghai, 200040 China; 8 Department of Emergency Neurology, Guiyang Medical University, Guiyang, 550004 China

**Keywords:** Type 2 diabetes, Cardio-cerebrovascular disease, Carbonic anhydraseIII, Autophagy

## Abstract

Type 2 diabetes mellitus (T2DM) is one of the most common chronic diseases among the elderly people. The T2DM increases the risk of cardio-cerebrovascular disease (CCD), and the main pathological change of the CCD is atherosclerosis (AS). Meanwhile, the carbonic anhydrases (CAs) are involved in the formation and progression of plaques in AS. However, the exact physiological mechanism of carbonic anhydrase III (CAIII) has not been clear yet, and there are also no correlation study between CAIII protein and T2DM with CCD. The 8-week old diabetic mice (db/db^−/−^ mice) and wild-type mice (wt mice) were feed by a normal diet till 32 weeks, and detected the carotid artery vascular opening angle using the method of biomechanics; The changes of cerebral cortex and myocardium were watched by the ultrastructure, and the autophagy were observed by electron microscope; The tissue structure, inflammation and cell injury were observed by Hematoxylin and eosin (HE) staining; The apoptosis of cells were observed by TUNEL staining; The protein levels of CAIII, IL-17, p53 were detected by immunohistochemical and Western Blot, and the Beclin-1, LC3, NF-κB were detected by Western Blot. All statistical analysis is performed using PRISM software. Compared with wt mice, db/db^−/−^ mice’ carotid artery open angle increased significantly. Electron microscope results indicated that autophagy in db/db^−/−^ mice cerebral cortex and heart tissue decreased and intracellular organelle ultrastructure were damaged. HE staining indicated that, db/db^−/−^ mice’ cerebral cortex and heart tissue stained lighter, inflammatory cells infiltration, cell edema were obvious, myocardial fibers were disorder, and myocardial cells showed different degrees of degeneration. Compared with wt mice, TUNEL staining showed that there was obviously increase in db/db^−/−^ mice cortex and heart tissue cell apoptosis. The results of immunohistochemistry and Western Blot indicated that CAIII, Beclin-1 and LC3II/I expression levels conspicuously decreased in cortex and heart tissue of db/db^−/−^ mice, and the expression level of IL-17, NF-κB and p53 obviously increased. The carotid artery’ vascular stiffness was increased and which was probably related with formation of AS in diabetic mice. And the autophagy participated in the occurrence and development of diabetic CCD. CAIII protein might somehow be involved in the regulation of autophagy probably through affecting cell apoptosis and inflammation, but the underlying mechanism remains to be further studied.

## Background

With the aging of population becoming more serious, health problems increase sharply with the changing of people’s life style, including diabetes mellitus, atherosclerosis, cardio-cerebrovascular disease (CCD), hypertension and neurodegenerative diseases etc.. (Vasilopoulos et al. 2014). Type 2 diabetes mellitus (T2DM) is one of the most common chronic diseases among the elderly, and the patients with T2DM worldwide will reach to 578.4 million by 2030 and 700.2 million by 2045 according to the 9th edition of IDF Diabetes Atlas (2019). Epidemiological studies showed that T2DM increased the risk of CCD by about 2–4 times, and even in the aggressive treatment of diabetes, the risk of myocardial infarction or stroke was seven to ten time that in individuals without diabetes (Gregg et al. 2014). In China, stroke is an important public health concern as the leading cause of morbidity and mortality (Yang et al. 2013), and the mortality rate of stroke in 2014 was 125.78 per 100,000 for urban residents and 151.91 per 100,000 in rural areas (Chen et al. 2017). Although CCD associated with diabetes is seriously harmful, its pathogenesis has not been fully figured out.

The pathogenesis and influencing factors of T2DM with CCD are extremely complicated, including genetic, insulin resistance, atherosclerosis (AS), hyperinsulinemia, and so on. AS is the most important factor. The cholesterol accumulation in the arterial wall causes the formation of plaque and vascular stenosis, which leads to the development of AS. When the plaque of AS ruptures, it may clog blood vessels and cause a variety of diseases, such as stroke, myocardial infarction, peripheral arterial thrombosis or another organ thrombosis. AS is a dynamic pathological process with complex mechanism, including inflammatory response and cellular senescence, which are closely related with autophagy dysfunction. It is a new field about autophagy in the development of AS. In recent research, it showed that autophagy may cause some specific diseases and play multiple roles in these diseases (Choi et al. 2013). Autophagy exists in eukaryotic cells and is used to degrade intracellular damaged organelles, abnormal proteins, DNA and other substances. And it can also reuse the amino acids, ribose and other products to maintain cell homeostasis. In normal physiological conditions, the level of autophagy is quite low. However under the conditions of hypoxia, ischemia, infection and other factors, autophagy will be enhanced and play a role of scavenging and prevents the activation of inflammatory responses in turn (Hubbard-Lucey et al. 2014). In the stress responses, a variety of cytokines are involved, such as IL-6, NF-κB and many other adipokines (Tavridou et al. 2011).

Carbonic anhydrases (CAs) are zinc-containing metalloproteinases, which are the main protein components with functions of accelerating the hydration and dehydration of carbon dioxide in red blood cells (Yoshimoto and Walde 2018). CAs exist in different tissues and are involved in many physiological processes, including gluconeogenesis, acid-base balance, adipogenesis, and calcification etc. (Supuran 2011). CAIII protein is a special member of CAs family, it involves in a variety of diseases, such as T2DM, myocardial injury, and skeletal muscle injury etc. (Lippi et al. 2008; Nishita et al. 1995). CAIII protein is a cytosolic enzyme with a relatively low activity of carbon dioxide hydratase and it may scavenge oxygen free radicals in vivo and protect cells from oxidative damage (Liu et al. 2012). The expression of CAs in AS are down-regulated, which indicates that CAs are involved in the occurrence and development of plaques (Yu 2010). In a recent study, the results indicated that the expression of CAIII protein was also down-regulated in Alzheimer’s disease (Kant et al. 2018).

However, the exact physiological mechanism of CAIII protein is not clear yet, and there are also no related research reports of the association between CAIII protein and CCD associated with T2DM. Therefore, the preliminary study of CAIII protein in diabetic animals with CCD and its correlation with autophagy dysfunction are the main purposes of our study.

## Materials and methods

### Animal grouping

Mice were purchased from the Model Animal Research Center of Nanjing University. We bought 40 8-week old mice which were divided into two groups by type: db/db^−/−^ mice and wt mice (20 mice per group). The approval number for experimentation with animals was 2018 HSJS-190. In order to observe the arteriosclerosis and CCD caused by diabetes mellitus, we fed 8-week old type 2 diabetic mice (db/db^−/−^ mice) and wild-type mice (wt mice) by a normal diet for 24 weeks, and they were free to eat and drink water till they are 32-week in experimental animal department of Fudan University Shanghai Medical College, with approval from the Ethics Committee for Animal Research.

### Vascular opening angle

The measurement of vascular opening angle was performed as described previously (Fung and Liu 1991). After the animal was sacrificed, the carotid artery was rapidly taken (0.5 cm), cut along the long axis of the artery, then the blood vessel rings were obtained. Placed the rings in oxygenated Krebs’ solution and equilibrated at 4 °C for 30 min. Next, the vascular ring was incised along the ventral and equilibrated at 4 °C for 10 min. Finally, scan the vascular ring with a scanner and measured the lateral angle at which the two ends of the vascular ring are connected, and that is the open angle.

### Transmission Electron microscopy

Transmission electron microscopy (TEM, PHILIPS CM-120, Netherlands) was performed as previously described (Gonzales et al. 2013). Tissues of the cortex and myocardium were perfused with 2.5% glutaraldehyde perfusate, fixed with 2.5% glutaraldehyde. Then fixation by fixative solution, dehydrated, embedded in paraffin, sliced and 3% uranyl acetate and lead citrate double staining. Finally, the sections were observed by CM-120 PHILIPS and photographed under transmission electron microscope.

### HE staining

Hematoxylin and eosin (HE) staining was performed as described previously (Guan et al. 2017). The paraffin-embedded sections of the cortex and myocardium were dewaxed and fixed with 95% ethanol for 20 min, washed twice with PBS, immersed in a hematoxylin staining solution for 2–3 min to stain the nuclear components. Then, the slices were re-washed with water and immersed in an eosin staining solution for 1 min to stain the cytoplasm.

### TUNEL staining

TUNEL staining was performed as described previously (Wei et al. 2017). The sections of the cortex and myocardium were incubated with protease K at room temperature for 30 min, washed with PBS twice, incubated with 50 μl TUNEL reaction mixture in a wet box at 37 °C for 60 min, then washed with PBS for three times. Next, the sections were incubated with 50 μl conversion agent-POD in the wet box at 37 °C for 30 min, washed with PBS for three times. Then, the color developing agent 3,3′-diaminobenzidine was dropped at room temperature for 10 min, and then the sections were washed with distilled water, followed by 1-min hematoxylin re-staining, dehydration and transparency. Finally, the sections were sealed via neutral gum and observed by microscope.

### Protein preparation

The right cortex and heart tissue proteins were extracted as the method described (Mlyniec et al. 2014a, b). Eight-month-old mice were sacrificed and the proteins from fresh cortex and heart tissue were prepared. The tissues were homogenized using RIPA buffer (20 mg of tissue with 200 μl of RIPA), and the tissue lysates were centrifuged at 12,000 rpm for 5 min and the supernatants were collected to determine the protein concentrations using a bicinchoninic acid (Beyotime Institute of Biotechnology, Shanghai, China).

### Western blotting

Western blotting with proteins of the cortex and myocardium was performed as described previously (Mlyniec et al. 2014a, b). To confirm equal loading of the samples on the gel, the membranes were re-probed with an antibody specific to GAPDH as an internal control. The specific primary antibodies used included rabbit polyclonal antibodies against CAIII (1:1000; abcam), Beclin-1 (1:200; Santa Cruz), LC3 (1:1000; abway), P53 (1:1000; abcam), IL-17 (1:1000; abcam), NF-κB (1:200; Santa Cruz), GAPDH (1:2000; Boster). Finally, the X-ray films were developed and fixed in a dark room.

### Immunohistochemistry

Immunohistochemistry was performed as described previously (Tu et al. 2017). Tissues samples of the cortex and myocardium were fixed with 4% polyformaldehyde, embedded in paraffin, cut into slices (thickness, 10 μm), dewaxed and hydrated. Then the slices were incubated with 3% hydrogen peroxide, washed with PBS, blocked with 10% normal goat serum at 37 °C for 30 min, incubated with rabbit polyclonal antibody for CAIII (1:200; Abcam), P53 (1:100; Abcam), IL-17 (1:1000; Abcam), in PBS containing 3% BSA overnight at 4 °C, followed by incubation with biotinylated secondary anti-rabbit antibody at 37°Cfor 45 min. Immunohistochemical staining followed by diaminobenzidine staining was subsequently performed. Finally, all sections were dehydrated sequentially with 75% ethanol for 5 min, 85% ethanol for 5 min, ethyl alcohol twice for every 5 min, and xylene for 6 min. They were then covered on slides for image analysis.

### Statistical analysis

All statistical analyses were performed using the GraphPad Prism 6.0 (Graph Pad; San Diego, CA, USA) and Excel (Microsoft Corporation; Redmond, WA, USA) software programs, and all data is represented as means ± standard error of the mean (SEM). The Western blot and tissue data was analyzed by student t-test. For all analyses, P-values less than 0.05 were considered significant.

## Results

### The vascular opening angle was increased in diabetic mice

**Fig. 1:**
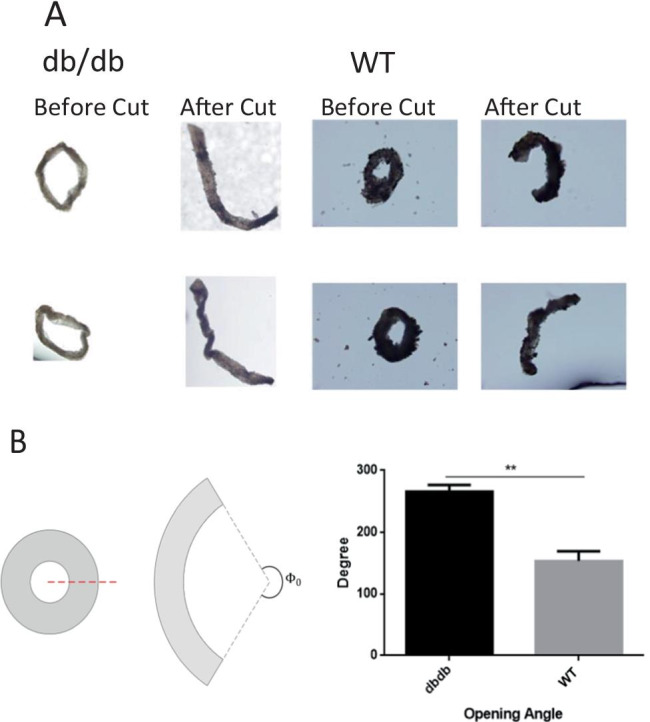
The carotid artery opening angles of db/db^−/−^ and WT were detected respectively, and the carotid artery vascular opening angle of db/db^−/−^ mice increased than WT mice (**, *P* < 0.01). N = 4

The vascular opening angle was considered to be a measurement of the residual strain of the vessel wall. As shown in Fig. 1, the left picture was the anterior vascular ring and the right one was the opening angle after cutting (Fig. 1, Φ0). The carotid artery opening angles in db/db^−/−^ and wt groups were detected respectively. Compared with wt mice, the carotid artery opening angle of db/db^−/−^ mice obviously increased (*P <* 0.01, Fig. 1). The result showed long-term diabetes might lead to increased vascular stiffness, which may be affected the structure, elasticity and remodeling of large blood vessels in turn.

### The brain and myocardium’ ultrastructure were damaged in diabetic mice

**Fig. 2:**
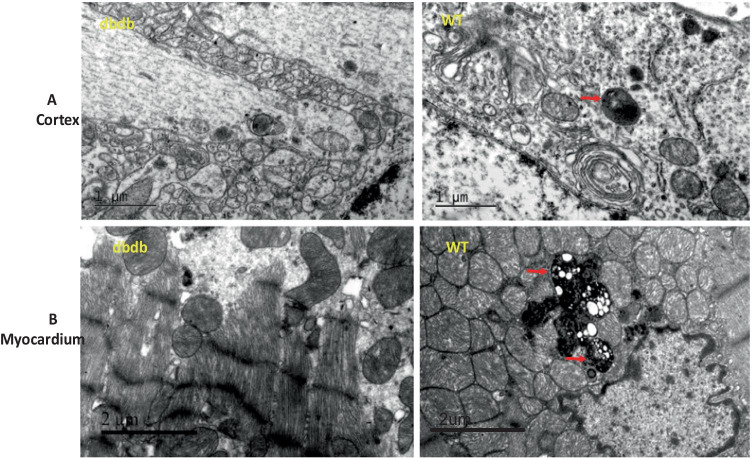
Compared with WT mice, the db/db^−/−^mice’ autolysosomes were significantly reduced, accompanied with nerve cells edema (**A**), and the mitochondria decreased and disorganized, myocardial fiber damaged (**B**). The arrows point to autolysosomes (Cortex, WT) or lipofuscin (Myocardium, WT), lipofuscin is composed of residual lipids digested by autolysosome. N = 4

Transmission electron microscopy was used to observe the ultrastructure of brain and myocardial tissues. The results showed that compared with wt mice, the db/db^−/−^ mice’ nerve cells were significantly edema, as well as the decrease of autolysosome formation, intracellular organelles and mitochondria (Fig. 2A). Compared with wt mice, the db/db^−/−^ mice’cardiac autolysosome were prominently reduced, mitochondria was decreased, endoplasmic reticulum was disorganized, and myocardial fiber was damaged even more (Fig. 2B). The above result showed that diabetes may cause the damage of tissues’ ultrastructure and autophagy disfunction.

### The cortex and myocardium in diabetic mice had obvious inflammation and cell damage

**Fig. 3:**
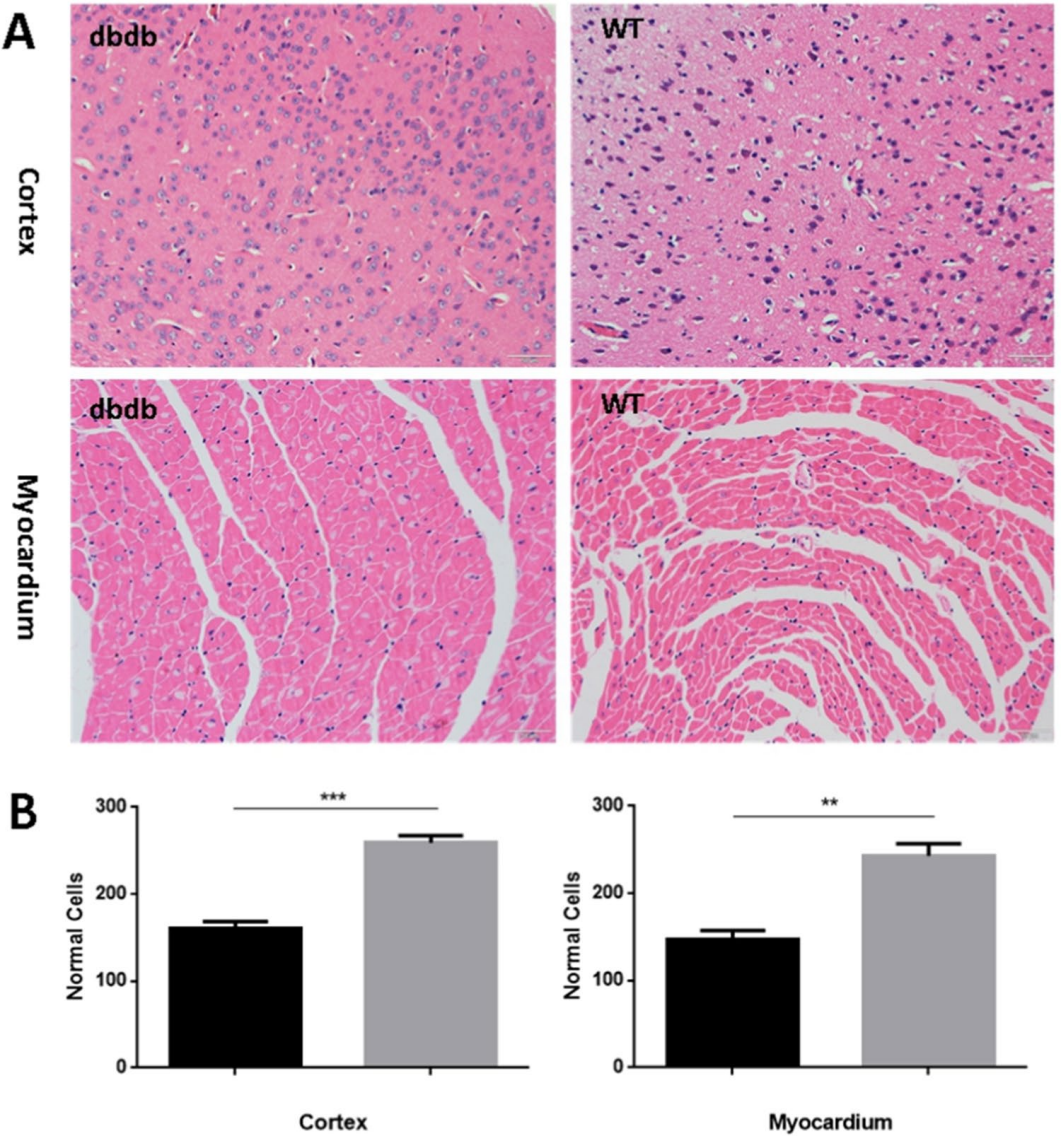
Hematoxylin and eosin (HE) staining showed that, the number of relative normal cells in cortex and myocardium of db/db^−/−^ mice were all significantly less than that in WT mice (Cortex, ***, *P* < 0.001; **, Myocardium, *P* < 0.01). Compared with WT mice, the cortex of db/db^−/−^ mice was shallowed lighter, microvascular and normal cells reduced, accompanied with inflammatory cells infiltration, significant edema and necrosis. And the cardiomyocytes in db/db^−/−^ mice were hypertrophy with irregular nuclei and different degrees of degeneration, and the myocardial fibers arranged disorder. N = 4

The pathological changes of cortex and myocardium were observed by Hematoxylin and eosin (HE) staining. HE staining showed that, the number of relative normal cells in cortex and myocardium of db/db^−/−^ mice were all significantly less than that in WT mice (all *P* < 0.01). As shown in Fig. 3A, the db/db^−/−^ mice’ cortex was shallowed lighter, microvascular and normal cells reduced, accompanied with inflammatory cells infiltration, significant edema and necrosis. In contrast, the wt mice’ cortex was shallowed deeper, with normal cell density, morphology and arrangement, and the interstitial cells were dense and uniform. The cardiomyocytes in db/db^−/−^ mice were hypertrophy with irregular nuclei and different degrees of degeneration, and the myocardial fibers arranged in disorder. While in the wt mice, the cardiomyocytes were slim and neat, and the myocardial fibers arranged densely (Fig. 3B).

### The apoptosis level of cortex and myocardium was increased in diabetic mice

**Fig. 4:**
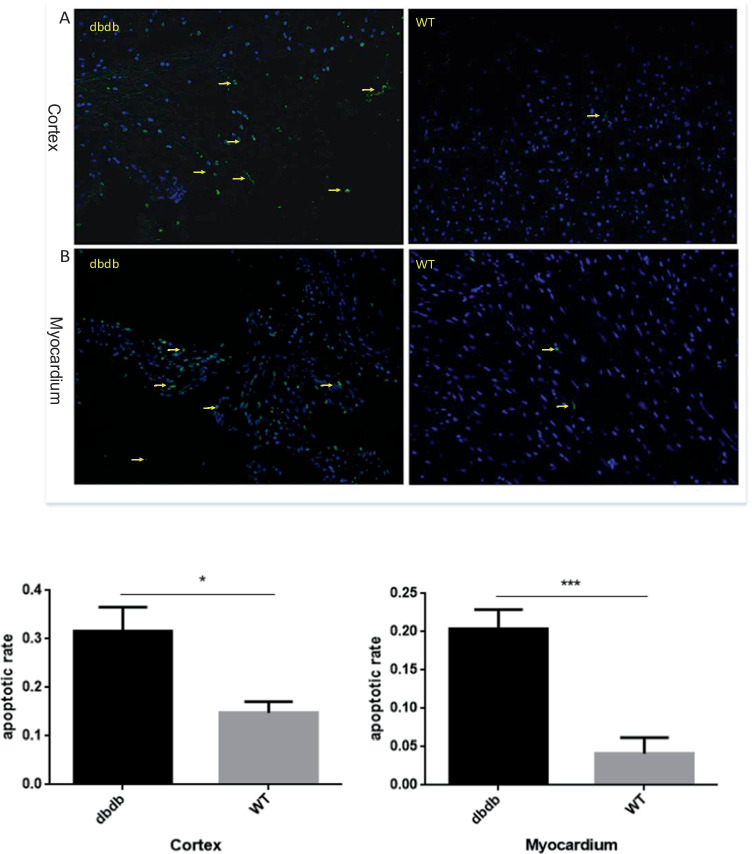
Tissue cells were stained by TUNEL to observe the apoptosis levels of cortex and myocardium, and The images of apoptosis were viewed at 200 x magnification. There were significant differences of the apoptotic cells in cortex and myocardium of db/db^−/−^ mice compared with WT mice (**A**: Cortex, *, *P* < 0.05; **B**: Myocardium, ***, *P* < 0.001). The arrows point to the apoptotic cells. N = 4

Tissue cells were stained by TUNEL to observe the apoptosis level of cortex and myocardium. As Fig. 4 shown, the apoptosis level of cortex and myocardium in diabetic mice increased in comparison to the wt mice (A: Cortex, *P* < 0.05; B: Myocardium, *P* < 0.001), and the results indicated that diabetes mellitus might induce excessive apoptosis and impaired function of tissue cells.

### The expression of CAIII protein was reduced in cortex and myocardium of diabetic mice

**Fig. 5:**
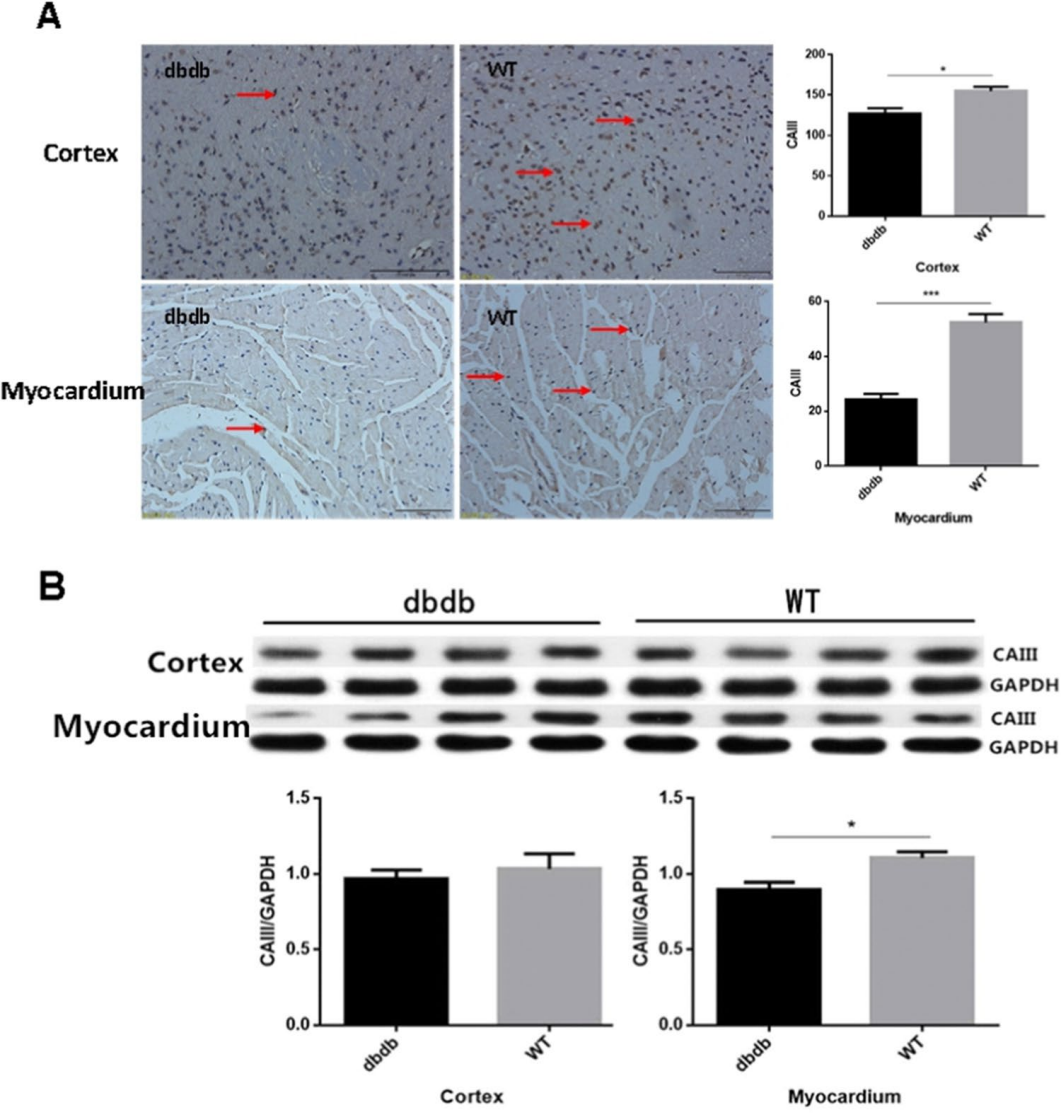
Immunohistochemical staining showed that the expression of CAIII protein obviously decreased in db/db^−/−^mice (**A**: Cortex *, *P* < 0.05; Myocardium ***, *P* < 0.001). Western Blotting indicated that, compared with WT group, the CAIII levels had a downward trend in cortex, While decreased significantly in myocardium of the db/db^−/−^group (**B**: Cortex, *P* > 0.05; Myocardium *, *P* < 0.05). The arrows point to the CAIII protein. N = 4

The expression of CAIII protein was assessed using immunohistochemistry and western blotting. The results showed that the expression of CAIII protein in cortex and myocardium of diabetic mice were obviously decreased (*P* < 0.05, Fig. 5A). Meanwhile, the results of western blotting confirmed that CAIII protein levels had a downward trend in cortex of the db/db^−/−^ group. In myocardium, the CAIII protein levels were decreased markedly in diabetic group compared with wt group (*P* < 0.05, Fig. 5B).

### The expression of Beclin-1 protein and the LC3-II/I ratio were reduced in cortex and myocardium of diabetic mice

**Fig. 6:**
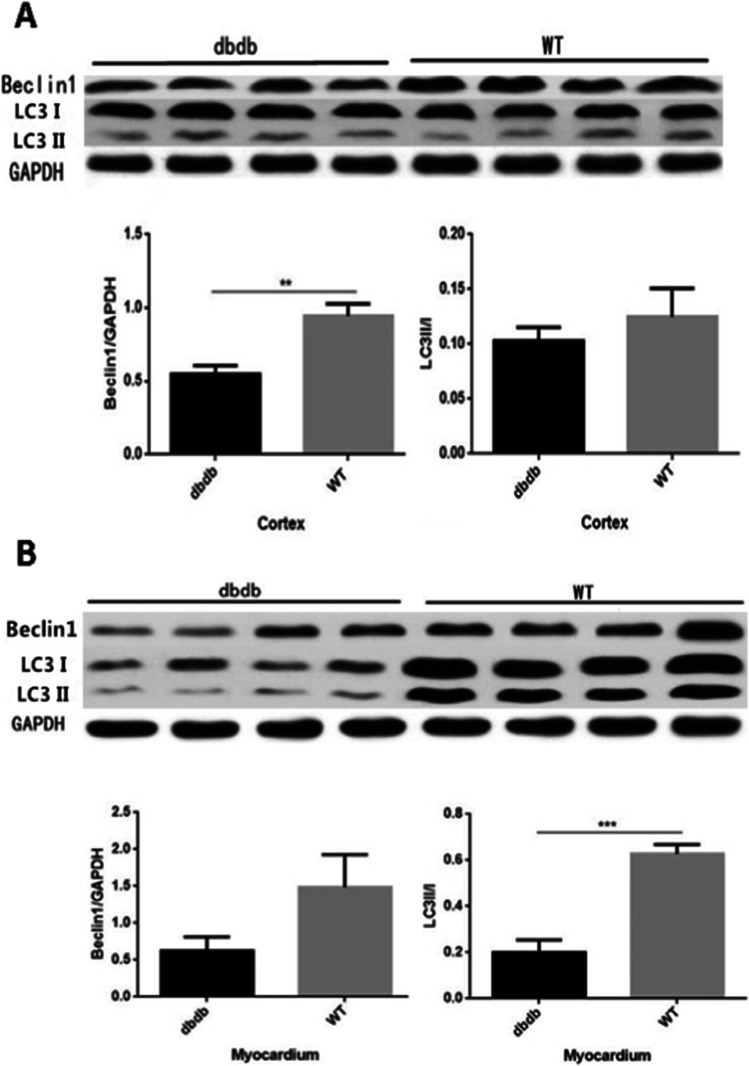
Western Blotting indicated that, the expression of Beclin-1 protein in cortex of db/db^−/−^group decreased significantly than WT (**A**: **, *P* < 0.01), while there was only a downward trend in expression of LC3II/I. In myocardium of db/db^−/−^group, both the expressions of Beclin-1 and LC3II/Iwere all reduced, and the difference of LC3II/Iwas significative (**B**: ***, *P* < 0.001). N = 4

As the western blotting results shown, the expression of Beclin-1 protein in cortex of diabetic group were decreased significantly than wt (*P* < 0.01, Fig. 6A). While the expression of LC3II/Ionly had a downward trend without difference (*P* > 0.05). In myocardium of diabetic group, both the expressions of Beclin-1 and LC3II/Iwere reduced, and the difference of LC3II/Iwas significative (*P* < 0.001, Fig. 6B). The results showed that the cortex and myocardium in diabetic mice may both have autophagy dysfunction.

### The expression of IL-17 and NF-κB were increased in cortex and myocardium of diabetic mice

**Fig. 7:**
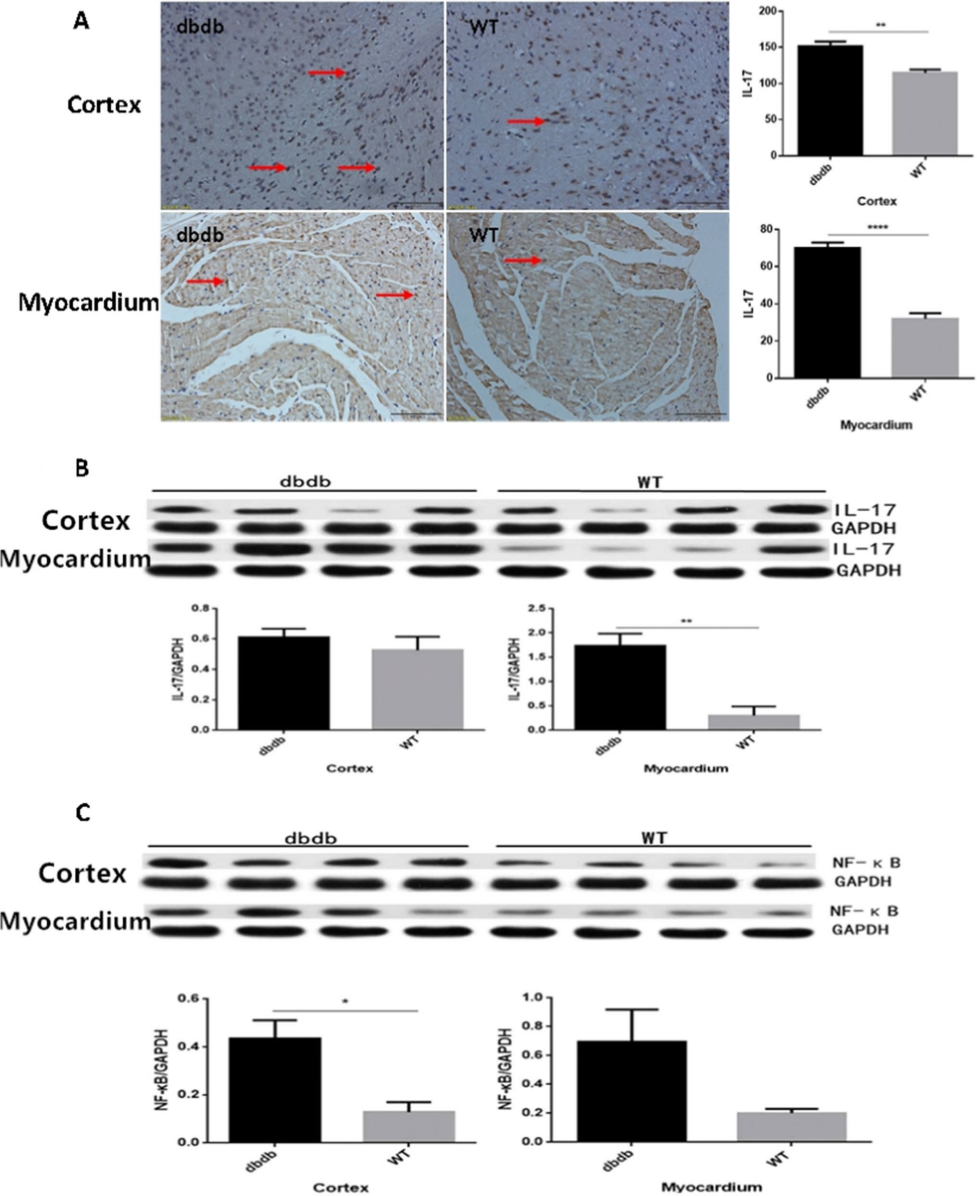
Immunohistochemical staining showed that the expressions of IL-17in cortex and myocardium of db/db^−/−^ group increased significantly than WT (**A**: Cortex **, *P* < 0.01; Myocardium ****, *P* < 0.0001). And the Western blotting results indicated that IL-17increased in db/db^−/−^ mice, especially in myocardium (**B**: **, *P* < 0.01). The expression of NF-κB protein in cortex of db/db^−/−^ group increased obviously than WT (**C**: *, *P* < 0.05). The expression of NF-κBin myocardium had a upward trend but without difference. The arrows point to the IL-17. N = 4

As the immunohistochemistry results show, the expression of IL-17 protein in cortex and myocardium of diabetic group were increased significantly than wt (*P* < 0.01 and *P* < 0.0001, Fig. 7A). And the results of western blotting showed that both the cortex and myocardium’ IL-17 protein were increased in diabetic mice, especially in myocardium (*P* < 0.01, Fig. 7B). The expression of NF-κB protein in cortex of diabetic group was increased obviously than wt (*P* < 0.05, Fig. 7C). Meanwhile, the expression of NF-κB protein in myocardium had a upward trend without difference. The results showed that there were obvious inflammatory responses in the cortex and myocardium of diabetic mice.

### The expression of p53 protein was increased in cortex and myocardium of diabetic mice

**Fig. 8:**
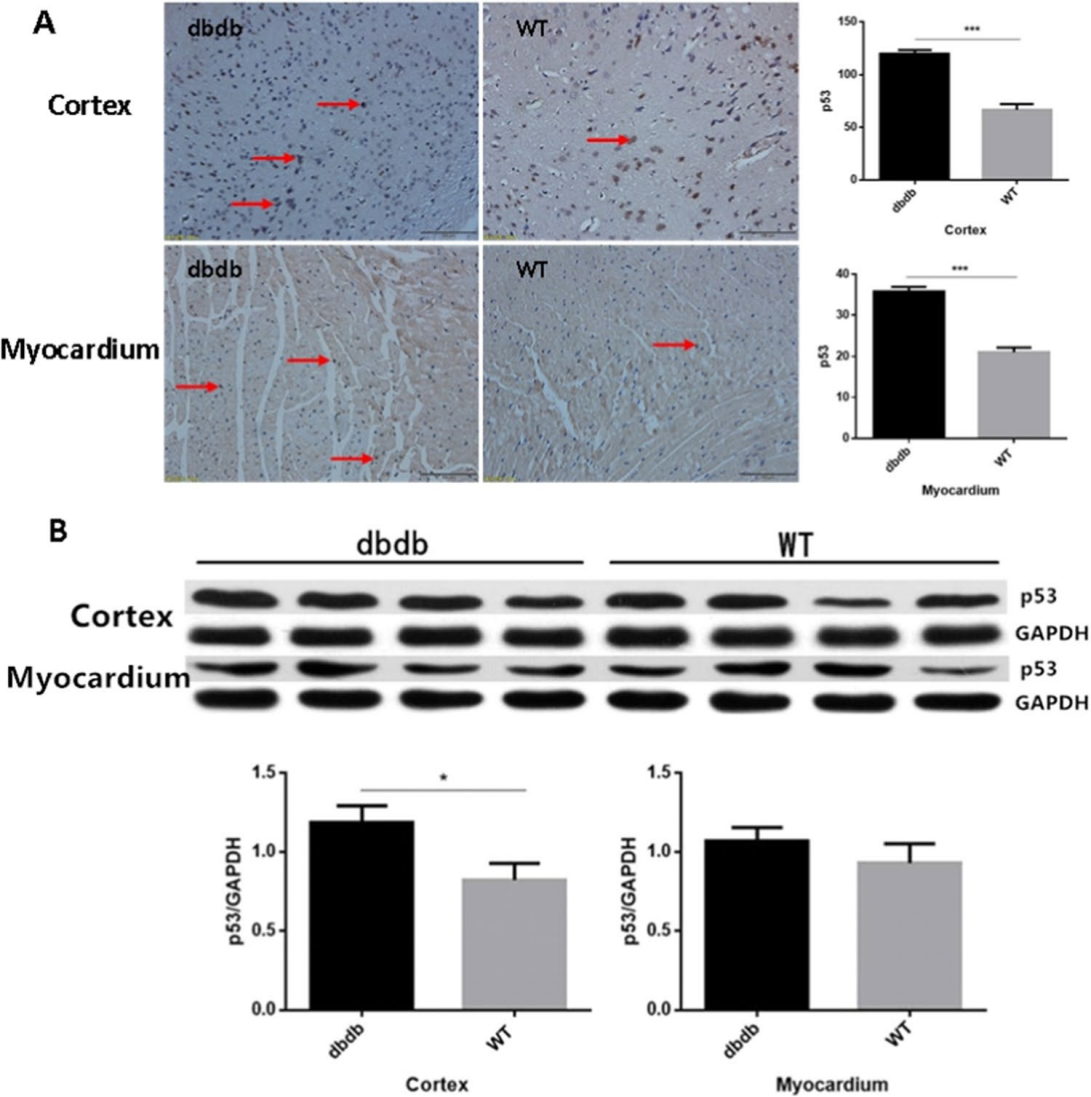
Immunohistochemical staining showed that the expression of p53 protein in cortex and myocardium of db/db^−/−^ group were all increased significantly than WT group (**A**: ***, *P* < 0.001). And the Western blotting results indicated that the p53 protein in cortex of db/db^−/−^ mice expressed more than WT mice (**B**: *, *P* < 0.05), while that in myocardium had no difference between two groups. The arrows point to the p53 protein. N = 4

As the immunohistochemistry results shown, the expression of p53 protein in cortex and myocardium of diabetic group was increased significantly than wt (*P* < 0.001, Fig. 8A). And the results of western blotting showed that the expression of p53 protein in diabetic mice was more than wt mice (*P* < 0.05), while there was no difference in myocardium (*P* > 0.05, Fig. 8B). The results showed that the apoptosis level of cortex and myocardium in diabetic mice might increase.

## Discussion

AS is the main pathological basis of T2DM combined with CCD. However, the mechanism of AS in diabetic patients are complicated and unclear. A variety of risk factors take part in the process of AS, many stress conditions can affect cell homeostasis in T2DM (Carneiro and Travassos 2013), and they break the dynamic balance between proliferation and apoptosis of the vascular endothelium which is a critical regulator of overall vascular health (Libby et al. 2013; Widlansky and Hill 2018). With the increasing of age, there are hypertrophy, hyperplasia and phenotype alterations of vascular smooth muscle cells (Intengan and Schiffrin 2001; Qiu et al. 2014; Ueno et al. 2000). Along with the opening angle changes, these processes have been described as adaptive responses to alterations in flow and pressure (Matsumoto et al. 1996). The vascular opening angle is a biomechanical indicator of zero-stress state of vascular, and it can be affected by the tissue proliferation and morphological. Previous studies showed that the minification of opening angle indicated a higher rate of death and a decrease in autologous contraction function of the vascular endothelial cells (Fung and Cowin 1993). Fung’s hypothesis of non-uniform remodeling states that if the inner wall grows more than the outer wall, the opening angle will increase; whereas if the outer wall grows more than the inner wall, the opening angle will decrease (Fung 1991). In this research, we found that the carotid artery’ opening angles in diabetic mice increased, and so speculated smooth muscle cells proliferated complicated with calcification during the progression of AS vascular stiffness increaseed, and vascular bed extension tension increased, resulting in the increase of vascular opening angle.

In the process of AS, the oxidative stress, which increasing in aging tissues due to decreased activity of antioxidant enzymes, is a key step (Bala et al. 2006; Tawakol and Jaffer 2018). As a special member of CAs, CAIII protein mainly presents in tissues characterized by a high oxygen consumption rate, such as skeletal muscle, liver, and brain, where they could participate in the processes of cell defense counteracting oxidative damage (Monti et al. 2017). CAIII protein not only regulate intracellular pH by delivering CO_2_ produced during cellular metabolism (Cote et al. 1999), and participate in cell defense processes counteracting oxidative damage, apoptosis and signal transduction (Monti et al. 2017), but also regulate the metabolism of intracellular energy. Meanwhile, some researches had found that the level of CAIII protein was almost unchanged after myocardial infarction thrombolysis, while myoglobin concentration increased significantly, so CAIII protein could be used in the differential diagnosis of acute myocardial infarction (Shiomi et al. 2013).

In T2DM, hyperglycemia and insulin resistance can increase the level of anaerobic conversion, lactic acid accumulation in red blood cells, and induce the level of CAIII protein protein decreasing, which will inhibit the binding of oxygen to hemoglobin and increase HbAlc (Speeckaert et al. 2014). The above reactions eventually lead to tissue hypoxia, inducing inflammation and oxidative stress in the blood vessels, and finally accelerate the development of T2DM and CCD. In our study, we found that the expression of CAIII protein was reduced in aging diabetic mice. In recently, some important results elucidating the physiological role of CA III protein in aging and aging-related processes have been obtained from a study on the nucleus pulposus phenotype, and the expression of CAIII was silenced of the nucleus pulposus cells, and the result showed high sensitivity to oxidative stress dependent apoptosis through caspase-3 activation. So it has been suggested that mechanisms regulating CAIII expression may represent novel therapeutic targets to reduce the negative effects of with aging (Silagi et al. 2018). Autophagy and apoptosis are interconnected. If stress persists and autophagy is not able to support cell survival in dying cells, apoptosis will be activated to induce an efficient elimination of cell debris (Kroemer et al. 2010). Meanwhile, as a marker of the apoptosis, the p53 protein has two effects on the process of autophagy (Tang et al. 2015): Firstly, it plays a role of transcription factor function to mediate autophagy in the nucleus; Secondly, it achieves its negative regulatory role of autophagy by the non-dependent transcriptional activity in the cytoplasm, the inhibition of AMPK activity and activation of mTOR pathway (Maiuri et al. 2010; Tasdemir et al. 2008). And there was also evidence that cells transfected with CAIII protein were protected from apoptosis induced by hydrogen peroxide (Raisanen et al. 1999).

As the core protein of autophagy, Beclin-1 not only stimulates or inhibits the occurrence of autophagy by regulating the formation of autophagy precursors, but also participates in the process of cell death and regulates the dynamic balance between autophagy and apoptosis (Kondo and Kondo 2006). The protein of LC3 is the hallmark of autophagy, and the expression of LC3–1 is downregulated while the LC3-II is opposite, and LC3-II is in direct proportion to the number of autophagic bodies (De Angulo et al. 2015). The autophagy works through two signal pathways: mTOR depended pathway and Beclin1/ClassIII PI3K3C depended pathway. Previous studies demonstrated that there were autophagy disfunctions mediated by Beclin-1 in brain tissues of diabetic mice, which might accelerate the development of cerebrovascular disease (Guan et al. 2016). Our results suggest that the expression of Beclin-1 is downregulated obviously in the pallium of diabetic mice, and the ratio of LC3II/I has a downward trend. So, there is autophagy dysfunction in brain tissue of diabetic mice.

Under the stress condition, the enhancement of autophagy can regulate the level of inflammation by inhibiting the formation of inflammatory complex and blocking the accumulation of inflammatory factors (Harris et al. 2011). However, if the autophagy dysfunction occurs, inflammatory responses cannot be regulated normally. IL-17 is a newly discovered key pro-inflammatory factor, which will be imbalanced in the aged and increase the incidence of cardiovascular disease, CCD and AS (van Leeuwen et al. 2009), and it can accelerate the formation of AS by inhibiting autophagy function and promote the expression of other inflammatory factors, such as NF-κB, which pathway has been revealed as a key molecular system involved in pathological brain inflammation (Cai and Liu 2012; Tavridou et al. 2011). Also the studies have suggested that NF-κB activation may be related to neuronal apoptosis, and it can induce cytotoxic products that exacerbate oxidative stress and promote apoptosis (Pahl 1999; Li et al. 2013).While the data from experiments using cell lines and animal models suggested that CA III protein might function to protect cells from oxidative damage. And the downregulation of antioxidant enzymes might be a trigger of autoimmune (Alver et al. 2011). And IL-17 also plays an important role in the pathogenesis of several autoimmune diseases (Yamada 2010). However, in our study, the apoptosis, oxidative stress and inflammation were whole increased, while CAIII protein was reduced in aging diabetic mice, so there might be more complex mechanisms waiting to be explored.

## Conclusion

In this research, we found that the carotid artery’ opening angles in diabetic mice increased, and suggested the carotid artery’ vascular stiffness was increased and which was probably related with formation of AS in diabetic mice.. Both the CA III protein and autophagy related proteins were decreased significantly in the brain and heart of db/db^−/−^ mice, suggesting that there may be internal relationship between CA III protein and autophagy. On one hand, the low-level of CA III protein may increase oxidative stress, local inflammatory response, tissue hypoxia and secretion of inflammatory factors, for example IL-17 and NF-κB. And the reaction process above may further inhibit autophagy. On the other hand, it may inhibit autophagy and induce cardiovascular and CCD by inducing the increase of p53 protein and promoting apoptosis. How does CA III protein regulate autophagy and participate in the development of AS and what signal pathways are used? Its objective truth and internal mechanism are not totally known yet.

In this study, we firstly found the decline of CAIII in the cortex and myocardium of db/db^−/−^mice, then confirm that the autophagy participated in the occurrence and development of diabetic CCD, and speculated that CAIII might somehow be involved in the regulation of autophagy probably through affecting cell apoptosis and inflammation. CAIII protein may be a potential intervention target to prevent or improve CCD associated with T2DM. but it still needs further in-depth systematic study, and our group are currently carrying on relevant research in this field in the future.

## Data Availability

Data sharing not applicable to this article as no data-sets were generated or analyzed in the current study.
